# Investigating the effectiveness of oral ketamine on pain, mood and quality of life in treatment resistant chronic pain

**DOI:** 10.3389/fpain.2023.1268985

**Published:** 2023-11-23

**Authors:** Sammy Al Mukhaizeem, Anurag Nasa, Dympna Waldron, Alex McDaid, Patrick J. Gorman, Molly Featherstone, Megan Barry, Paul Murphy, Hugh Gallagher, Shrijit Nair, Michael O’Connor, Linda Kelly, Emma O'Hora, Roisin Dolan, Siaghal Mac Colgain, Jack McGrath, Stephane Blouin, Elena Roman, Laura Gaffney, Darren William Roddy, Kirk John Levins

**Affiliations:** ^1^ Department of Anaesthesia, St Vincent’s University Hospital, Dublin, Ireland; ^2^ School of Medicine, College of Health and Agricultural Sciences, University College Dublin, Dublin, Ireland; ^3^ Department of Psychiatry, Education and Research Centre, Royal College of Surgeons, Beaumont Hosptial, Dublin, Ireland; ^4^ Department of Palliative Medicine, Saolta Hospitals Group, Galway University Hospital, Galway, Ireland; ^5^ Department of Medicine, University of Galway, Galway, Ireland; ^6^ SPIRIT (St Vincent’s Pain Institute: Research, Innovation and Therapies), St Vincent’s University Hospital, Dublin, Ireland; ^7^ Department of Plastic Surgery, St Vincent’s University Hospital, Dublin, Ireland; ^8^ National Maternity Hospital, Dublin, Ireland

**Keywords:** oral, ketamine, chronic, pain, treatment-resistant

## Abstract

**Introduction:**

Chronic pain is defined as pain lasting longer than 3 months. This often causes persistent emotional distress and functional disability that is refractory to conventional treatments. Emerging evidence suggests that oral Ketamine therapy may have a specific role in managing treatment-resistant chronic pain. This study aimed to assess the effectiveness of oral ketamine within a tertiary chronic pain management clinic.

**Methods:**

This study was a clinic-based retrospective descriptive study of 79 patients with a broad range of chronic pain diagnoses and treated with oral ketamine over a period up to 12 years. Changes in pain, mood and quality of life (QoL) were assessed using a numerical pain severity score, the Brief Pain Inventory (BPI), the Public Health Questionnaire (PHQ-9) and American Chronic Pain Association Quality of Life (QoL) scale.

**Results:**

73 patients were accessible for follow-up (mean daily dose and treatment duration were 193.84 mg and 22.6 months respectively). Pain scores decreased (*p* < 0.0001) on both numerical scores (41.6% decrease) and BPI scoring (mean decrease 2.61). Mood improved (*p* < 0.0001) across both PHQ-9 and BPI measurements. Patients also reported less difficulty with daily activities and improved QoL. The most common adverse reaction was drowsiness (21.9%), with 30.1% reporting no adverse reactions from Ketamine.

**Discussion:**

This work adds to the growing body of evidence that under the supervision of a pain specialist, oral ketamine therapy may be a safe, tolerable and effective treatment for chronic pain conditions which have not responded to other management options. Further research is required to produce a more accurate understanding of its chronic use.

**Key message:**

This real-world study shows that patients being treated with oral ketamine for chronic pain report decreased severity of pain, improved mood and increased quality of life across all conditions.

## Introduction

Chronic pain is pain that persists for longer than 3 months. The ICD-11 has 7 subcategories of chronic pain, categorized as primary or secondary (1). It defines primary chronic pain as pain present in at least one anatomical region that results in emotional distress or functional disability and does not fit the definition of another chronic pain condition. Fibromyalgia and complex regional pain syndrome (CRPS) are examples of these chronic pain conditions. Secondary chronic pain is defined as a symptom of an underlying condition. It can be cancer-related, post-surgical, musculoskeletal, visceral, neuropathic or orofacial (2). The prevalence of chronic pain conditions approaches 20% worldwide (3) increasing with age (4). It impacts quality of life significantly (5) and is the leading cause of years lived with disability (6). Chronic pain is also associated with decreased life expectancy (7). Living with chronic pain increases the chances of developing depression and anxiety-like symptoms as well as the diagnosis of depression and anxiety disorders (8–11). Management of chronic pain is complex and often involves a combination of interventional, pharmacological, psychological and physical therapies (12).

The N-methyl-D-aspartate receptor (NMDAR) is found on neural structures involved in nociception such as primary afferent neurons and spinal dorsal horn neurons (13). It is thought to play a role in development and persistence of chronic pain (14). These excitatory glutamatergic receptors facilitate the transmission of afferent pain signals. In chronic pain states, these receptors are upregulated in the spinal cord through long-term potentiation causing central sensitization (15). This results in patients experiencing increased pain sensitivity (hyperalgesia), pain from non-painful stimuli (allodynia) and even spontaneous pain (16). The non-competitive NMDAR antagonist ketamine has been shown to reduce allodynia and hyperalgesia in chronic pain patients and may potentially reverse the neuronal changes seen in central sensitization (17).

Ketamine (originally referred to as CI-581) was first synthesised in 1962 as a shorter-acting, and therefore safer, alternative to Phencyclidine (CI-395) (18). Both the anaesthetic and analgesic properties of ketamine are primarily due to NMDAR antagonism (19). It also acts on nicotinic, muscarinic and opioid receptors and increases ambient noradrenaline (17).

Ketamine can be administered through intravenous, intramuscular, intrarectal, oral, or intranasal routes (13). Epidural administration of ketamine is typically avoided as it enters systemic circulation. It is a lipophilic molecule with an extensive volume of distribution that undergoes extensive first pass hepatic metabolism. As such, it has a low oral bioavailability, estimated at 10%–20% (20). In adults, ketamine has an elimination half-life of 2–3 h (21) while it’s metabolite Norketamine persists 5 h after Ketamine administration (22). This causes accumulation of Norketamine which itself is implicated in the sustained analgesic effect after Ketamine has been eliminated (22).

The use of Ketamine for the treatment of chronic pain has largely been limited to those with pain resistant to other interventional options (23). There is insufficient evidence to indicate greater efficacy across administration routes of Ketamine for chronic pain. It has been shown that with IV administration, the analgesic effect of ketamine is maintained for steady-state plasma concentrations greater than 100–160 ng/ml (24). However, due to Norketamine, effective analgesic concentrations with oral Ketamine are lower at 40 ng/ml (25). Adverse effects such as psychedelic phenomena, anxiety and paranoia typically occur at higher plasma concentrations than the analgesic effect (26, 27).

This dose-dependent correlation with adverse reactions may be explained by Ketamine's action primarily on NMDA receptors at low doses, with actions on opioid, dopaminergic and monoaminergic receptors occurring at higher doses (28). Using oral formulations to achieve the lowest effective plasma concentration for analgesia seems desirable. When used for treatment resistant depression (TDD), both oral and IV ketamine have been suggested to have similar efficacy (29). The oral route of administration is a cheaper, more convenient and personally acceptable administration method for choice of maintenance treatment when compared to other routes (30).

Despite the increasing use of ketamine for chronic pain management, there remains a paucity of widespread evidence to support its effectiveness, particularly over the longer-term (31). Most studies have involved small numbers of patients, restricted diagnostic categories and short follow-up. This study aims to explore the effectiveness of oral ketamine across multiple chronic pain conditions in a retrospective study investigating pain scores, mood and quality of life (QoL) in an outpatient setting.

## Methods

### Study design

This was a retrospective descriptive study of 73 patients with treatment-resistant chronic pain. Ethical approval was sought prior to commencement.

### Selection criteria

The criteria for this study were as follows:

• Aged >18 years
• English Fluency
• Chronic pain diagnosis of any type
• Prescribed Oral Ketamine for the treatment of chronic pain after unsatisfactory pain relief from all other available interventional options suitable to the patient

### Setting

This study was conducted at the above outpatient pain clinic from June 2020 to June 2021. This is the largest tertiary-level pain clinics in Ireland. Ketamine is offered as a management option to patients with severe intractable chronic pain. It is only offered in the form of an oral solution (10 mg/ml), which patients self-administer following a training session with a specialist nurse. Ketamine is prescribed directly from this pain clinic and full prescription list was generated through a review of current and archived prescription pads.

Patients were contacted by letter with a detailed patient information leaflet (PIL) and consent form. A follow-up phone call from a member of the study team explained the PIL and consent form. Following consent, each patient was contacted to arrange a suitable time to complete the questionnaires. Both ‘pre’ and ‘post’ Ketamine responses were recorded during a single interview.

### Reduce bias

To reduce bias, interviews were conducted by study team members who fulfilled the following criteria: never met the patient, never involved in the patient’s care, not licensed to prescribe ketamine in Ireland and appropriately trained in the required questionnaires.

### Data collected

Interviews were performed by phone and involved applying questionnaires and scales to patients’ current situation as well as recollecting how their pain, mood and QoL were prior to starting ketamine. All data was anonymised on collection, transcribed and stored in a secure database prior to analysis. Information was elicited with:

• A standard numerical pain scale (0–10).
• Difficulty with activities of daily living (ADLs) due to pain. Difficulty was ranked as follows: 1 none, 2 mild, 3 moderate, 4 severe.
• The patient health questionnaire (PHQ-9) (32). This consists of 9 questions, screening for the common signs and symptoms of depression. The depression severity is scored as follows: 0–4 none, 5–9 mild, 10–14 moderate, 15–19 moderately severe, 20–27 severe. A PHQ-9 of ≥10 has a sensitivity and specificity of 88% for major depression.
• The brief pain inventory (BPI) (33). This questionnaire explores the impact of pain using a numerical rating (0–10) for worst, least, average, and current pain intensity and the degree that pain interferes with general activity, mood, walking ability, normal work, relations with other persons, sleep, and enjoyment of life. This has been shown to hold validity across cultures and languages and can be used for all types of chronic pain (34).
• American Chronic Pain Association Quality of Life Scale. This is a one-item scale measuring QoL specifically in pain populations (35). With this scale patients can succinctly describe their ability to complete ADLs measuring from 0 (no functioning) to 10 (normal daily functioning).
• A list of medications while on oral Ketamine therapy and any pain related procedures ever.

### Statistical analysis

All statistical analysis of the data was performed on the IBM SPSS-27 software (https://www.ibm.com/analytics/us/en/technology/spss/). Data was pre-processed before data analysis, involving inspection and outlier winsorization of the data. The data from responses in the questionnaires were not normally distributed, so a non-parametric approach was preferred throughout the analysis. Descriptive statistics were performed. Following this, the Mann–Whitney *U* test was performed to compare findings between the responses for before and after initiation of Ketamine therapy.

## Results

### Participants

**Figure 1:**
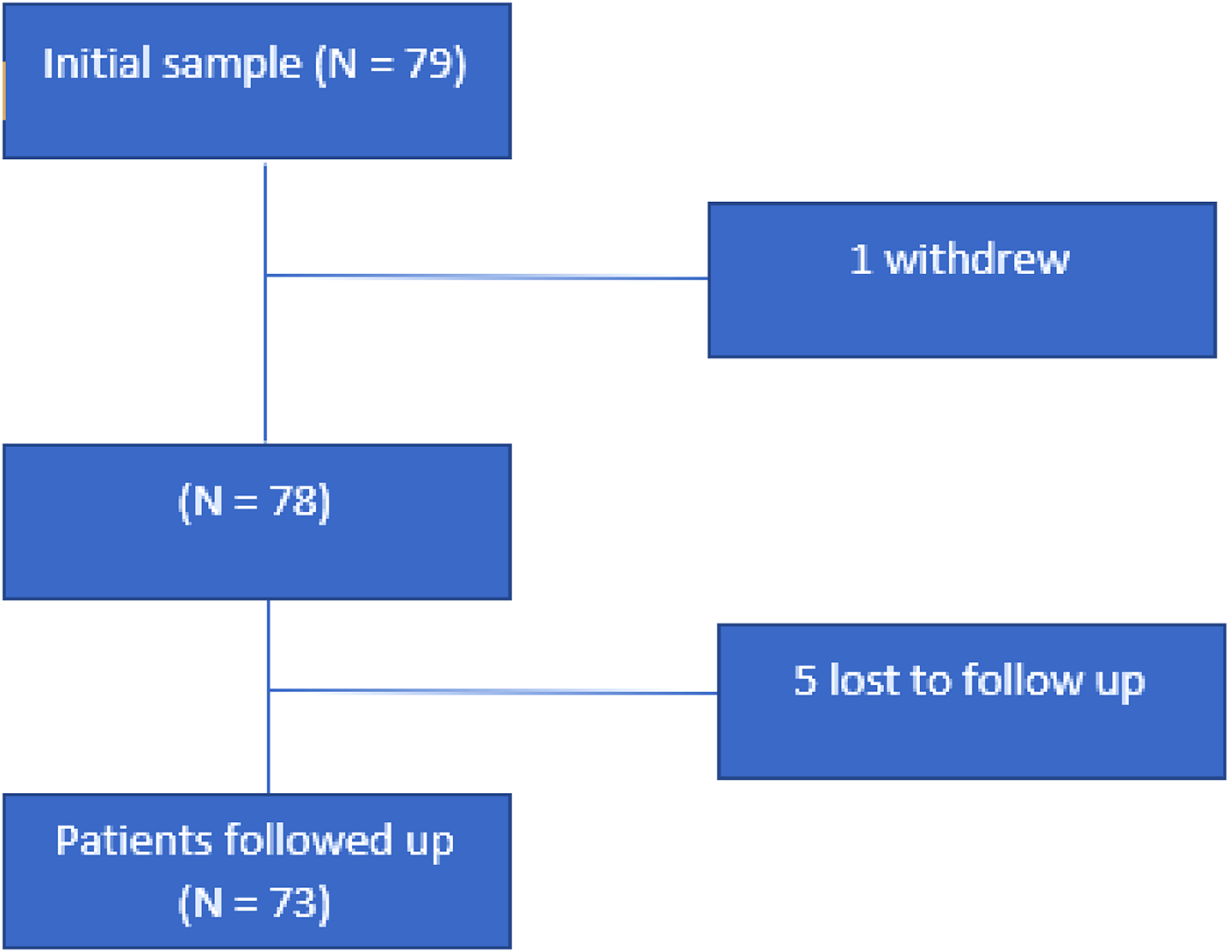
Flowchart of participant cohort. N, number.

From June 2020 to June 2021, a total of 79 patients were recruited from St Vincent’s University Hospital’s Pain Management Clinic. One patient withdrew, and five others could not be reached for follow-up (Figure 1). Among the remaining 73 patients, 80.8% (*n* = 59) were female. The average age was 49 ± 12 years, and the average daily dose of Ketamine was 193.84 ± 176.84 mg (Table 1). The average treatment duration was 22.6 ± 26.6 months, ranging from 1 week to over 12 years, with a median of 1 year. At follow-up, 5.5% (*n* = 4) of patients had discontinued ketamine. For the “post” ketamine section of the questionnaire, patients were asked to recall the period immediately after completing ketamine therapy.

**Table 1:** Sex, age and cumulative daily dose of ketamine for participants.

Baseline characteristic	*n*	%	Mean	SD	Min. Value	Max. Value
Gender						
Male	14	19.2				
Female	59	80.8				
Age						
(In years)			48.56	12.36	20	84
Cumulative Ketamine dose						
(Mg/Day)			193.84	1	0	1,500

Mg, milligrams; *N*, number; SD, standard deviation.

The patients included in this study had a range of chronic pain diagnoses (Table 2). The most common were fibromyalgia 21.9% (*n* = 16), peripheral neuropathy 17.8% (*n* = 13), chronic back/neck pain 16.4% (*n* = 12) and complex regional pain syndrome (CRPS) 13.7% (*n* = 10).

**Table 2:** Chronic pain diagnoses.

Chronic pain diagnosis	*N*	%
Meralgia paraesthesia	1	1.4
Fibromyalgia	16	21.9
Chondrosarcoma	1	1.4
Chronic back pain	12	16.4
Complex regional pain syndrome	10	13.7
MS	3	4.1
Trigeminal neuralgia	1	1.4
Radiculopathy	3	4.1
Migraine	2	2.7
Peripheral neuropathy	13	17.8
Somatic pain	3	4.1
Visceral pain	6	8.2
Hidradenitis suppurativa	1	1.4
Chemotherapy induced neuropathy	1	1.4
Total	73	100

N, number of participants.

### Interviews and questionnaires

The 10-point numerical pain scale decreased by 3.67 points following ketamine therapy. 39.7% (*n* = 29) reported that the nature of their pain had changed. The PHQ-9 score decreased from 15.07 to 9.97 following Ketamine treatment (Table 3).

**Table 3:** Scores for pain, quality of life, difficulty with activities of daily living (ADLs) and patient health questionnaire-9 (PHQ-9) before and after commencing ketamine.

	*n*	Mean	SD	Min.	Max.	*P*-value	% Change
Pain score							
Pre-Ketamine	73	8.82	1.25	6	10		
Post-Ketamine	73	5.15	2.09	1	9	<0.0001	−41.61
Quality of life							
Pre-Ketamine	72	3.88	2.35	0	9		
Post-Ketamine	73	6.31	2.03	2	10	<0.0001	+62.63
Difficulty with ADLs							
Pre-Ketamine	71	3.08	0.98	1	4		
Post-Ketamine	69	2.26	0.9	1	4	<0.0001	−26.62
PHQ-9							
Pre-Ketamine	73	15.07	6.45	0	27		
Post-Ketamine	73	9.97	5.42	1	21	<0.0001	−33.84

*N*, number of participants; SD, standard deviation.

BPI-scoring patients reported improvements across all 10-point domains (Table 4). Maximum, minimum and average pain were all reported to decrease (2.91, 2.16 and 2.61 respectively). The impact of pain also showed a general decrease across all BPI domains; general activity (1.23), mood (3.58), enjoyment of life (3.26), relationships (2.65), sleep (2.95), walking ability (3.58) and normal work (2.89). Patients reported experiencing less difficulty regarding activities of daily living, and QoL was found to improve by 2.43 points (Table 3).

**Table 4:** Brief pain inventory (BPI) scores before and after commencing ketamine.

	*n*	Mean	SD	Min.	Max.	*P*-value	% Reduction
Highest pain severity							
Pre-Ketamine	73	9.55	0.73	8	10		
Post-Ketamine	73	6.64	2.64	0	10	<0.0001	−30.47
Lowest pain severity							
Pre-Ketamine	72	5.79	2.67	0	10		
Post-Ketamine	73	3.63	2.49	0	10	<0.0001	−37.31
Average pain severity							
Pre-Ketamine	73	7.43	1.82	3	10		
Post-Ketamine	73	4.82	2.31	0	10	<0.0001	−35.13
Impact of pain on							
General activity							
Pre-Ketamine	72	8.62	1.64	5	10		
Post-Ketamine	71	7.39	2.86	0	10	0.013	−14.27
Mood							
Pre-Ketamine	73	7.62	2.61	1	10		
Post-Ketamine	70	4.04	3.06	0	10	<0.0001	−46.98
Walking ability							
Pre-Ketamine	71	7.62	2.61	1	10		
Post-Ketamine	73	4.04	3.06	0	10	<0.0001	−14.27
Normal work							
Pre-Ketamine	73	8.15	1.96	4	10		
Post-Ketamine	73	5.27	3.04	0	10	<0.0001	−35.34
Relations with other people							
Pre-Ketamine	73	6.68	3.28	0	10		
Post-Ketamine	73	4.03	2.99	0	10	<0.0001	−39.67
Sleep							
Pre-Ketamine	73	7.9	2.36	2	10		
Post-Ketamine	73	4.95	3.48	0	10	<0.0001	−37.34
Enjoyment of life							
Pre-Ketamine	73	8.03	1.9	3	10		
Post-Ketamine	73	4.78	2.84	0	10	<0.0001	−40.47

*N*, number of participants; SD, standard deviation.

All reported differences were significant on Mann-Whitney U testing with a significance of <0.0001, except BPI general activity, which was significant at 0.013 (Table 4).

### Adverse reactions

Various adverse reactions were reported (Table 5). Drowsiness was the most reported adverse reaction at 21.92% (*n* = 16). Other adverse reactions include nausea 12.33% (*n* = 9), memory impairment 10.96% (*n* = 8) and dizziness 8.22% (*n* = 6). 30.14% (*n* = 22) of patients reported having no adverse reactions to Ketamine**.**

**Table 5:** Adverse reactions to ketamine.

	*n*	%
Adverse reaction		
None	22	30.14
Memory impairment	8	10.96
Impact on mood	1	1.37
Nausea & vomiting	9	12.33
Impact on sleep	4	5.48
Dizziness	6	8.22
Drowsiness	16	21.92
Hallucinations	2	2.74
Cystitis	3	4.11
Headache	2	2.74
Numbness	1	1.37
Hot Flushes	2	2.74
Diarrhoea	1	1.37

*N*, number of participants.

### Concurrent medications and procedures

Table 6 shows the concurrent medications used by patients while on ketamine. Opioids were the most common medication, being used by 60.3% (*n* = 44) of patients. Other common medications include amitriptyline 26.03% (*n* = 19), pregabalin 23.29% (*n* = 17) and gabapentin 16.44% (*n* = 12). 5.48% (*n* = 4) reported taking no concurrent medications whilst undergoing ketamine therapy.

**Table 6:** Concurrent medications.

Medication	*N*	%
Opioids	44	60.27
Pregabalin	17	23.29
Amitriptyline	19	26.03
Mirtazapine	1	1.37
NSAIDs	17	23.29
Paracetamol	22	30.14
Tizanidine	4	5.48
Gabapentin	12	16.44
Naltrexone	8	10.96
Esomeprazole	6	8.22
Naloxone	4	5.48
Duloxetine	5	6.85
Methadone	1	1.37
Lamotrigine	1	1.37
Flunarizine	1	1.37
Citalopram	1	1.37
Methotrexate	1	1.37
Lidocaine	2	2.74

*N*, number of participants.

Table 7 shows procedures either before or during ketamine therapy. These include nerve blocks 42.47% (*n* = 31), spinal cord stimulators 24.66% (*n* = 18), steroid injections 10.96% (*n* = 8) and Botox injections 9.59% (*n* = 7). 13.7% (*n* = 10) of patients reported having no previous procedures for the alleviation of pain.

**Table 7:** Proceudres underwent by patients for the purpose of pain relief.

Procedure	*N*	%
Nerve block	31	42.47
Spinal cord stimulator	18	24.66
Discectomy	3	4.11
Rhizotomy	5	6.85
Botox injection	7	9.59
Laminectomy	2	2.74
Spinal fusion	4	5.48
Trigger point injection	3	4.11
PRP injection	1	1.37

*N*, number of participants; PRP, platlet-rich plasma.

## Discussion

This retrospective cohort study of 73 patients with various chronic pain conditions showed decreased pain, improved mood, improved QoL and increased ease with undertaking ADLs when treated with oral ketamine.

Decreases in both the numerical pain scale and BPI pain scores were found (Tables 3, 4). Clinically signficant decreases in chronic pain have been suggested at 27.9–33% (36, 37). A 5-year retrospective study of patients with intractable chronic pain found 66% of patients reported a ≥25% reduction in baseline pain with 44% of patients reporting ≥50% reduction when treated with oral Ketamine (27). Our results of a decrease of 3.67 points (41.6%) overall in the numerical pain score and similar reductions in the BPI maximum (30.5%), minimum (37.3%) and average pain scores (35.1%) are in keeping with previous findings. 39.7% (*n* = 29) reported that the nature of their pain had improved since starting oral ketamine.

The PHQ-9 decreased from 15.07 to 9.97 following oral Ketamine treatment (Table 3). A change of 5 or more points on the PHQ-9 is regarded as a clinically significant improvement in mood (39) from moderately severe (score > 15) to mild/moderate depression symptoms (between 9 and 14). The PHQ-9 is a valid measure of depressive severity when used both in patients with diagnosed major depression and the general (non-diagnosed) population (32). The PHQ-9 results are reflected in the BPI mood scores with a reduction of 46.9% in depression symptoms. Ketamine has recently found traction in the treatment of resistant depression (40). Chronic pain is thought to involve similar brain changes to those found in depression (41–45), with patients up to four times more likely to suffer from depression or anxiety (46) and are twice as likely to die by suicide (2). Chronic pain patients have shown improvement in depressive symptoms using 150 mg of oral Ketamine daily (47). Whether our improvement in mood was secondary to improved pain, or through a different primary mechanism remains uncertain.

Ketamine's interaction with the dorsal diencephalic conduction system (DDCS) may help explain the improvements seen in mood. The DDCS consists of three key structures; the habenula (medial and lateral), the stria medullaris, and the fasciculus retroflexus (48). This highly conserved pathway links the monoaminergic brainstem and the basal forebrain. Most importantly, its central component modulates the lateral habenula which acts as a hub before transmitting signals to brainstem modulatory areas (49). This makes the lateral habenula imperative for the integration of emotional, cognitive, sensory and motor processing to ultimately influence value-based decision-making and motivational processing (50). It has been widely recognised, using fMRI resolutions, that the lateral habenula plays a significant role as an ‘anti-reward center’ in the mechanism of addiction and major depression (51). Ketamine has been shown to inhibit burst firing from the lateral habenula via NMDA receptor antagonism (52). This is suggested to be the primary mechanism of Ketamine's antidepressant properties.

BPI-scoring reported improvements across all domains including general activity, mood, enjoyment of life, relationships, sleep, walking ability and normal work (Table 4). Reflecting these improvement, patients reported less difficulty with activities of daily living and QoL (Table 3). A detrimental effect of living with chronic pain is clear with poorer QoL (53). Furthermore, it impacts work-related outcomes including productivity and absences (54). When self-reported pain intensity is reduced by over 50%, chronic pain sufferers consistently report improvements in work and QoL (54). Unfortunately, up to 79% of chronic pain patients report their pain as being ineffectively managed (55).

Oral ketamine has been reported to be generally well tolerated when used in treatment of both chronic pain and depression with dizziness, visual disturbance and loss of balance being the most common reported effects (56). Patients in these studies report these side effects were mild and did not pose a major burden on treatment maintenance. Nearly a third of patients in our study reported having no adverse effects from oral Ketamine. Side effects reported (Table 5) were largely mild. Furthermore, these effects may not be solely attributed to ketamine as concurrent pharmacological intervention was being utilised (Table 6).

Conversely, poor tolerability with high rates of adverse reactions have also been reported. In a study investigating subcutaneous ketamine for cancer pain the number needed to harm (NNH) was just 6 (57). In comparison, the number needed to treat (NNT) was 25. However, rapid dose escalation (from 100 mg to 300 mg to 500 mg) may account for the high rate of adverse reactions encountered in this study, unlike the usual gradual increases in an outpatient chronic pain setting. Our study suggests that relatlively lower doses of Ketamine (mean < 200 mg) in a carefully monitored chronic pain specialist outpatient department may result in fewer adverse effects.

The growing body of evidence to support Ketamine's safety and tolerability is based on short time-limited term studies. Unfortunately, evidence to support its safety with chronic use is lacking (58). Case reports suggest severe ulcerative cystitis (59) and hepatotoxicity (60) may develop with chronic ketamine use. Animal models suggest ketamine can induce hyperphosphorylated tau in the cerebral cortex, a hallmark of Alzheimer’s disease (61). Further research is needed provide a more accurate understanding of the effects associated with chronic usage.

Ketamine is a controlled medication in most countries. Prevalence of illicit recreational use in the general population is thought to be 0.8% (62). In the clinical setting, data is lacking as to the extent of illicit usage and diversion of prescriptions, particularly when used to treat chronic pain (63). A subset of a case series assessing the use of repeated IV ketamine infusions for up to 126 weeks to treat depression made no reports of addiction, dependence or abuse (64). Similar findings were seen in a 48 week trial of intranasal ketamine for depression (65).

The opioid crisis in the United States and elsewhere is a stark warning on the dangers of drug overpresciption (66). Therefore we must exercise caution when prescribing oral Ketamine to treat chronic pain, and limit its use to those with severe intractable chronic pain who have explored all other suitable interventions.

### Limitations and strengths

As this is a single-centre tertiary care study based in one country (Ireland), our results may not readily generalisable to other centres or countries (67). However, multicentre-controlled studies of Ketamine treatment have shown similar improvements between centres and countries (68). The retrospective nature of our interviews may be susceptible to recall bias. Being an open-label study and lacking a control group, we acknowledge the possibility that some patients may have experienced a placebo effect of pain reduction (69). Furthermore, patients continued to have various concurrent pharmacological and procedural interventions whilst using oral ketamine. We therefore accept that these may have contributed to the improvements seen.

Patients’ views of pain can recalibrate over time. This recalibration, known as ‘Response Shift’ (RS), suggests that if a subjective symptom such as pain is measured using an outcome measure without incorporating RS, any true change may not be captured (70). Until we have pain measurement tools incorporating RS with scientific reliability/validity, then the patients’ word remains the best outcome measure (71, 72).

All patients were included regardless of pain type, severity, sex, medications, procedures, comorbid diagnoses, and disability level. Rather than focusing on specific pain syndromes with a predefined level of disability, this study demonstrates the overall effectiveness of oral ketamine with improvements in pain, mood and Qol with an ecological validity that may be useful for a busy tertiary pain clinic where chronic pain of all types and severity is encountered. This study contributes to the evidence for Ketamine as a useful treatment in a tertiary pain clinic for all types and severity of chronic pain and associated disabilities.

## Conclusion

This retrospective study found that chronic pain patients reported improvements in pain, mood and quality of life (QoL) with oral ketamine in a real world outpatient setting.

## Data Availability

The original contributions presented in the study are included in the article/Supplementary Material, further inquiries can be directed to the corresponding author.

## References

[1] World Health Organization. International statistical classification of diseases and related health problems. 11th revision. (2021) 6 (Retrieved September 2018). 10.2471/BLT.25.294650 PMC13531092 42683092

[2] Tang NK Crane C. Suicidality in chronic pain: a review of the prevalence, risk factors and psychological links. Psychol Med. (2006) 36(5):575–86. 10.1017/S0033291705006859 16420727

[3] Breivik H Collett B Ventafridda V Cohen R Gallacher D. Survey of chronic pain in Europe: prevalence, impact on daily life, and treatment. Eur J Pain. (2006) 10(4):287–333. 10.1016/j.ejpain.2005.06.009 16095934

[4] Johannes CB Le TK Zhou X Johnston JA Dworkin RH. The prevalence of chronic pain in United States adults: results of an internet-based survey. J Pain. (2010) 11(11):1230–9. 10.1016/j.jpain.2010.07.002 20797916

[5] Dueñas M Ojeda B Salazar A Mico JA Failde I. A review of chronic pain impact on patients, their social environment and the health care system. J Pain Res. (2016) 9:457–67. 10.2147/JPR.S105892 27418853 PMC4935027

[6] Vos T Flaxman AD Naghavi M Lozano R Michaud C Ezzati M Years lived with disability (YLDs) for 1160 sequelae of 289 diseases and injuries 1990–2010: a systematic analysis for the global burden of disease study 2010. Lancet. (2012) 380(9859):2163–96. 10.1016/S0140-6736(12)61729-2 23245607 PMC6350784

[7] Smith D Wilkie R Uthman O Jordan JL McBeth J. Chronic pain and mortality: a systematic review. PLoS One. (2014) 9(6):e99048. 10.1371/journal.pone.0099048 24901358 PMC4047043

[8] Lerman SF Rudich Z Brill S Shalev H Shahar G. Longitudinal associations between depression, anxiety, pain, and pain-related disability in chronic pain patients. Psychosom Med. (2015) 77(3):333–41. 10.1097/PSY.0000000000000158 25849129

[9] Goldenberg DL. The interface of pain and mood disturbances in the rheumatic diseases. Semin Arthritis Rheum. (2010) 40(1):15–31. 10.1016/j.semarthrit.2008.11.005 19217649

[10] Bair MJ Robinson RL Katon W Kroenke K. Depression and pain comorbidity: a literature review. Arch Intern Med. (2003) 163(20):2433–45. 10.1001/archinte.163.20.2433 14609780

[11] Murphy F Nasa A Cullinane D Raajakesary K Gazzaz A Sooknarine V Childhood trauma, the HPA axis and psychiatric illnesses: a targeted literature synthesis. Front Psychiatry. (2022) 13:748372. 10.3389/fpsyt.2022.748372 35599780 PMC9120425

[12] Center for Substance Abuse T. SAMHSA/CSAT treatment improvement protocols. Managing chronic pain in adults with or in recovery from substance use disorders. Rockville (MD): Substance Abuse and Mental Health Services Administration (US) (2012). 22514862

[13] Mion G Villevieille T. Ketamine pharmacology: an update (pharmacodynamics and molecular aspects, recent findings). CNS Neurosci Ther. (2013) 19(6):370–80. 10.1111/cns.12099 23575437 PMC6493357

[14] Lee M Silverman SM Hansen H Patel VB Manchikanti L. A comprehensive review of opioid-induced hyperalgesia. Pain Physician. (2011) 14(2):145–61. 10.36076/ppj.2011/14/145 21412369

[15] Niesters M Martini C Dahan A. Ketamine for chronic pain: risks and benefits. Br J Clin Pharmacol. (2014) 77(2):357–67. 10.1111/bcp.12094 23432384 PMC4014022

[16] Sigtermans MJ van Hilten JJ Bauer MCR Arbous SM Marinus J Sarton EY Ketamine produces effective and long-term pain relief in patients with complex regional pain syndrome type 1. Pain. (2009) 145(3):304–11. 10.1016/j.pain.2009.06.023 19604642

[17] Culp C Kim HK Abdi S. Ketamine use for cancer and chronic pain management. Front Pharmacol. (2021) 11:599721. 10.3389/fphar.2020.599721 33708116 PMC7941211

[18] Domino EF. Taming the ketamine tiger. Anesthesiology. (2010) 113(3):678–84. 10.1097/ALN.0b013e3181ed09a2 20693870

[19] Wan LB Levitch CF Perez AM Brallier JW Iosifescu DV Chang LC Ketamine safety and tolerability in clinical trials for treatment-resistant depression. J Clin Psychiatry. (2015) 76(3):247–52. 10.4088/JCP.13m08852 25271445

[20] Clements JA Nimmo WS Grant IS. Bioavailability, pharmacokinetics, and analgesic activity of ketamine in humans. J Pharml Sci. (1982) 71:539–42. 10.1002/jps.2600710516 7097501

[21] Domino EF Domino SE Smith RE Domino LE Goulet JR Domino KE. Ketamine kinetics in unmedicated and diazepam premedicated subjects. Clin Pharmacol Ther. (1984) 36:645–53. 10.1038/clpt.1984.235 6488686

[22] Malinovsky JM Servin F Cozian A Lepage JY Pinaud M. Ketamine and norketamine plasma concentrations after IV, nasal and rectal administration in children. Br J Anaesth. (1996) 77:203–7. 10.1093/bja/77.2.203 8881626

[23] Mangnus TJP Bharwani KD Stronks DL Dirckx M Huygen FJPM. Ketamine therapy for chronic pain in The Netherlands: a nationwide survey. Scand J Pain. (2021) 22(1):97–105. 10.1515/sjpain-2021-0079 34432970

[24] Clements JA Nimmo WS Grant IS. Bioavailability, pharmacokinetics, and analgesic activity of ketamine in humans. J Pharm Sci. (1982) 71:539–42. 10.1002/jps.2600710516 7097501

[25] Grant IS Nimmo WS Clements JA. Pharmacokinetics and analgesic effects of IM and oral ketamine. Br J Anaesth. (1981) 53:805–10. 10.1093/bja/53.8.805 7272143

[26] Bowdle TA Radant AD Cowley DS Kharasch ED Strassman RJ Roy-Byrne PP. Psychedelic effects of ketamine in healthy volunteers: relationship to steady-state plasma concentrations. Anesthesiology. (1998) 88:82–8. 10.1097/00000542-199801000-00015 9447860

[27] Hartvig P Valtysson J Lindner KJ Kristensen JD Karlsten R Gustafsson LL Central nervous system effects of subdissociative doses of (S)-ketamine are related to plasma and brain concentrations measured with positron emission tomography in healthy volunteers. Clin Pharmacol Ther. (1995) 58:165–73. 10.1016/0009-9236(95)90194-9 7648766

[28] Bell RF Kalso EA. Ketamine for pain management. Pain Rep. (2018) 3(5):e674. 10.1097/PR9.0000000000000674 30534625 PMC6181464

[29] Irwin SA Iglewicz A Nelesen RA Lo JY Carr CH Romero SD Lloyd LS. Daily oral ketamine for the treatment of depression and anxiety in patients receiving hospice care: a 28-day open-label proof-of-concept trial. J Palliat Med. (2013) 16:958–65. 10.1089/jpm.2012.0617 23805864 PMC3717203

[30] Andrade C. Oral ketamine for depression, 2: practical considerations. J Clin Psychiatry. (2019) 80:19f12838. 10.4088/JCP.19f12838 30997961

[31] Jonkman K van de Donk T Dahan A. Ketamine for cancer pain: what is the evidence? Curr Opin Support Palliat Care. (2017) 11(2):88–92. 10.1097/SPC.0000000000000262 28306568

[32] Kroenke K Spitzer RL Williams JB. The PHQ-9: validity of a brief depression severity measure. J Gen Intern Med. (2001) 16(9):606–13. 10.1046/j.1525-1497.2001.016009606.x 11556941 PMC1495268

[33] Cleeland CS Ryan KM. Pain assessment: global use of the brief pain inventory. Ann Acad Med Singap. (1994) 23(2):129–38. PMID: . 8080219

[34] Tan G Jensen MP Thornby JI Shanti BF. Validation of the brief pain inventory for chronic nonmalignant pain. J Pain. (2004) 5(2):133–7. 10.1016/j.jpain.2003.12.005 15042521

[35] Mason VL Skevington SM Osborn M. A measure for quality of life assessment in chronic pain: preliminary properties of the WHOQOL-pain. J Behav Med. (2009) 32(2):162–73. 10.1007/s10865-008-9187-y 19057988

[36] Farrar JT Young JP Jr LaMoreaux L Werth JL Poole MR. Clinical importance of changes in chronic pain intensity measured on an 11-point numerical pain rating scale. Pain. (2001) 94(2):149–58. 10.1016/S0304-3959(01)00349-9 11690728

[37] Farrar JT Portenoy RK Berlin JA Kinman JL Strom BL. Defining the clinically important difference in pain outcome measures. Pain. (2000) 88(3):287–94. 10.1016/S0304-3959(00)00339-0 11068116

[38] Marchetti F Coutaux A Bellanger A Magneux C Bourgeois P Mion G. Efficacy and safety of oral ketamine for the relief of intractable chronic pain: a retrospective 5-year study of 51 patients. Eur J Pain. (2015) 19(7):984–93. 10.1002/ejp.624 25381898

[39] Löwe B Unützer J Callahan CM Perkins AJ Kroenke K. Monitoring depression treatment outcomes with the patient health questionnaire-9. Med Care. (2004) 42(12):1194–201. 10.1097/00005650-200412000-00006 15550799

[40] Riggs LM Gould TD. Ketamine and the future of rapid-acting antidepressants. Annu Rev Clin Psychol. (2021) 17:207–31. 10.1146/annurev-clinpsy-072120-014126 33561364 PMC8170851

[41] Levins KJ Drago T Roman E Martin A King R Murphy P Magnetic resonance spectroscopy across chronic pain disorders: a systematic review protocol synthesising anatomical and metabolite findings in chronic pain patients. Syst Rev. (2019) 8(1):338. 10.1186/s13643-019-1256-5 31882014 PMC6935150

[42] Roddy D O'Keane V. Cornu ammonis changes are at the core of hippocampal pathology in depression. Chronic Stress (Thousand Oaks). (2019) 3:2470547019849376. 10.1177/2470547019849376 32440594 PMC7219935

[43] Roddy D Kelly JR Farrell C Doolin K Roman E Nasa A Amygdala substructure volumes in major depressive disorder. Neuroimage Clin. (2021) 31:102781. 10.1016/j.nicl.2021.102781 34384996 PMC8361319

[44] Roddy DW Kelly JR Drago T Raajakesary K Haines M O’Hanlon E. Neurobiochemistry alterations associated with major depression: a review of translational magnetic resonance spectroscopic studies. In: Kim Y-K Amidfar M, editors. Translational research methods for major depressive disorder. New York, NY: Springer US (2022). p. 265–309.

[45] Drago T O’Regan PW Welaratne I Rooney S O’Callaghan A Malkit M A cmprehensive regional neurochemical theory in depression: a protocol for the systematic review and meta-analysis of 1H-MRS studies in major depressive disorder. Syst Rev. (2018) 7(1):158. 10.1186/s13643-018-0830-6 30309391 PMC6182786

[46] Gureje O Von Korff M Simon GE Gater R. Persistent pain and well-being: a world health organization study in primary care. JAMA. (1998) 280(2):147–51. 10.1001/jama.280.2.147 9669787

[47] Jafarinia M Afarideh M Tafakhori A Arbabi M Ghajar A Noorbala AA Efficacy and safety of oral ketamine versus diclofenac to alleviate mild to moderate depression in chronic pain patients: a double-blind, randomized, controlled trial. J Affect Disord. (2016) 204:1–8. 10.1016/j.jad.2016.05.076 27317968

[48] Roman E Weininger J Lim B Barry D Tierney P O’Hanlon E Untangling the dorsal diencephalic conduction system: a review of structure and function of the stria medullaris, habenula and fasciculus retrofexus. Brain Struct Funct. (2020) 225:1437–58. 10.1007/s00429-020-02069-8 32367265

[49] Carpenter MB. Core text of neuroanatomy. 4th ed. Baltimore: Williams & Wilkins (1991).

[50] Gardon O Faget L Chu Sin Chung P Matifas A Massotte D Kiefer BL. Expression of mu opioid receptor in dorsal diencephalic conduction system: new insights for the medial habenula. Neuroscience. (2014) 277:595–609. 10.1016/j.neuroscience.2014.07.053 25086313 PMC4164589

[51] Loonen AJM. Role of neuroglia in the habenular connection hub of the dorsal diencephalic conduction system. Neuroglia. (2023) 4(1):34–51. 10.3390/neuroglia4010004

[52] Yang Y Cui Y Sang K Dong Y Ni Z Ma S Ketamine blocks bursting in the lateral habenula to rapidly relieve depression. Nature. (2018) 554(7692):317–22. 10.1038/nature25509 29446381

[53] Hadi MA McHugh GA Closs SJ. Impact of chronic pain on patients’ quality of life: a comparative mixed-methods study. J Patient Exp. (2019) 6(2):133–41. 10.1177/2374373518786013 31218259 PMC6558939

[54] Patel AS Farquharson R Carroll D Moore A Phillips CJ Taylor RS The impact and burden of chronic pain in the workplace: a qualitative systematic review. Pain Pract. (2012) 12(7):578–89. 10.1111/j.1533-2500.2012.00547.x 22462774

[55] Bekkering GE Bala MM Reid K Kellen E Harker J Riemsma R Epidemiology of chronic pain and its treatment in The Netherlands. Neth J Med. (2011) 69(3):141–53. PMID: . 21444943

[56] Schoevers RA Chaves TV Balukova SM aan het Rot M Kortekaas R. Oral ketamine for the treatment of pain and treatment-resistant depression. Br J Psychiatry. (2016) 208:108–13. 10.1192/bjp.bp.115.165498 26834167

[57] Hardy J Quinn S Fazekas B Plummer J Eckermann S Agar M Randomized, double-blind, placebo-controlled study to assess the efficacy and toxicity of subcutaneous ketamine in the management of cancer pain. J Clin Oncol. (2012) 30(29):3611–7. 10.1200/JCO.2012.42.1081 22965960

[58] Short B Fong J Galvez V Shelker W Loo CK. Side-effects associated with ketamine use in depression: a systematic review. Lancet Psychiatry. (2018) 5(1):65–78. 10.1016/S2215-0366(17)30272-9 28757132

[59] Shahani R Streutker C Dickson B Stewart RJ. Ketamine-associated ulcerative cystitis: a new clinical entity. Urology. (2007) 69(5):810–2. 10.1016/j.urology.2007.01.038 17482909

[60] Lo RS Krishnamoorthy R Freeman JG Austin AS. Cholestasis and biliary dilatation associated with chronic ketamine abuse: a case series. Singapore Med J. (2011) 52(3):e52–5. PMID: . 21451916

[61] Wu ZG Chen F Wu H Chen JX Wei QT Fu YQ Urinary metabonomics of rats with ketamine-induced cystitis using GC-MS spectroscopy. Int J Clin Exp Pathol. (2018) 11(2):558–67. PMID: ; PMCID: . 31938141 PMC6958055

[62] Office for National Statistics (ONS). Drug Misuse in England and Wales: year ending March 2020. (2020).

[63] Sassano-Higgins S Baron D Juarez G Esmaili N Gold M. A review of ketamine abuse and diversion. Depress Anxiety. (2016) 33(8):718–27. 10.1002/da.22536 27328618

[64] Wilkinson ST Katz RB Toprak M Webler R Ostrof RB Sanacora G. Acute and longer-term outcomes using ketamine as a clinical treatment at the Yale Psychiatric Hospital. J Clin Psychiatry. (2018) 79:17m11731. 10.4088/JCP.17m11731 30063304 PMC6296748

[65] Wajs E Aluisio L Holder R Daly EJ Lane R Lim P Esketamine nasal spray plus oral antidepressant in patients with treatment-resistant depression: assessment of long-term safety in a phase 3, open-label study (SUSTAIN-2). J Clin Psychiatry. (2020) 81:19m12891. 10.4088/JCP.19m12891 32316080

[66] Ayoo K Mikhaeil J Huang A Wąsowicz M. The opioid crisis in North America: facts and future lessons for Europe. Anaesthesiol Intensive Ther. (2020) 52(2):139–47. 10.5114/ait.2020.94756 32419434 PMC10176520

[67] Bellomo R Warrillow SJ Reade MC. Why we should be wary of single-center trials. Crit Care Med. (2009) 37(12):3114–9. 10.1097/CCM.0b013e3181bc7bd5 19789447

[68] Corriger A Voute M Lambert C Pereira B Pickering G. Ketamine for refractory chronic pain: a 1-year follow-up study. Pain. (2022) 163(4):690–701. 10.1097/j.pain.0000000000002403 34252909

[69] Cepeda MS Berlin JA Gao CY Wiegand F Wada DR. Placebo response changes depending on the neuropathic pain syndrome: results of a systematic review and meta-analysis. Pain Med. (2012) 13(4):575–95. 10.1111/j.1526-4637.2012.01340.x 22390269

[70] Mannion DE Gilmartin JJ McInerney V Molony K Basquille E Waldron D. Exploring the innate human potential for positive adaptation in the face of impending mortality: is there a response shift in subjective quality of life over time in a group of patients with lung cancer receiving palliative treatment? Inter J Innov Res Med Sci. (2020) 5(05):160–9. 10.23958/ijirms/vol05-i05/880

[71] McInerney DV. An intervention using quality of life and symptom information as a clinical tool in patients with advanced cancer. Inter J Innov Res Med Sci. (2019) 4(08):517–24. 10.23958/ijirms/vol04-i08/740

[72] McInerney VB Waldron D Mannion E. Using individual quality-of-life information as a clinical tool in patients with advanced cancer. J Clin Oncol. (2015) 33(29_suppl):91. 10.1200/jco.2015.33.29_suppl.91

